# A novel poly (vinyl alcohol)/poly (ethylene glycol) scaffold for tissue engineering with a unique bimodal open-celled structure fabricated using supercritical fluid foaming

**DOI:** 10.1038/s41598-019-46061-7

**Published:** 2019-07-02

**Authors:** Ping Liu, Wenhua Chen, Cuihua Liu, Ming Tian, Pengju Liu

**Affiliations:** 1 Children’s Hospital Affiliated to Zhengzhou University, Henan Provincial Children’s Hospital, Zhengzhou Children’s Hospital, Zhengzhou, 450000 China; 2 State Key Laboratory of Polymer Materials Engineering, Polymer Research Institute of Sichuan University, Chengdu, 610065 China

**Keywords:** Tissues, Materials science

## Abstract

In this study, a novel poly (vinyl alcohol) (PVA)/poly (ethylene glycol) (PEG) scaffold was carefully designed via thermal processing and subsequent supercritical fluid (SCF) foaming. Interestingly, a bimodal open-celled structure with interconnected networks was successfully created in the plasticized PVA (WPVA)/PEG scaffold. Large cells were produced from the nucleation sites generated in the PVA phase during rapid depressurization, while plenty of small pores generate in the cell walls of the big cells. The formation mechanism of this cellular structure was studied by considering the various phase morphologies and the diffusion behaviour of the carbon dioxide (CO_2_) in individual phases. In addition, the intermolecular interactions of the WPVA/PEG blend were studied using X-ray diffraction and FTIR analysis. The results demonstrate that various types of hydrogen bonds among the hydroxyl groups on the PVA chains, PEG and water molecules are formed in the blend system. The realization of thermoplastic foaming of the PVA/PEG blend benefits from the interactions of complexation and plasticization between water and PEG molecules. The SEM images also revealed that L929 fibroblast cells were able to attach and spread on surfaces of the WPVA/PEG samples. Thus the WPVA/PEG scaffold with unique bimodal cellular structure is nontoxic and favours the attachment and proliferation of cells, making it promising for use as the candidate for tissue engineering applications.

## Introduction

Recently, environmentally friendly and biodegradable polymer foams have attracted increasing attention from academic and industrial researchers. This is because these porous materials have been widely developed for drug delivery application and as the scaffold for tissue engineering^1–3^. As a representative of this family, poly (vinyl alcohol) (PVA) is not only biodegradable but can also be produced via non-petroleum routes, which is an alternative to the exhaustion of petroleum resources. For tissue engineering applications, it is generally required that the porous materials have good mechanical strength, biocompatibility and biodegradability. The interconnected network allows the easy diffusion of nutrients and wastes, while the hierarchical pore structure is critical in seeding and adhesion of cells^4,5^. PVA is a typical polar polymer with multi-hydroxyl groups and shows good comprehensive performance, including generally superior mechanical and thermal properties compared to polyolefin, with favourable biocompatibility, and excellent barrier properties, thus making it an ideal candidate for a variety of tissue engineering applications, such as bone, cartilage, and the aortic heart valve^6,7^. In addition, PVA has an uncomplicated chemical structure and the abundant pendant hydroxyl groups provide various possibilities for PVA modification by simple chemical reaction. However, because of the narrow gap between the melting point and decomposition temperature of PVA, to date, the realization of PVA foam that has been reported in most literatures is confined to solution-based methods, which are complex and inefficient. Thus, the development of a new strategy for producing such structured PVA foam is still a great challenge and is urgently needed.

Over the past few decades, supercritical fluid (SCF) technique has developed into an advantageous method in terms of preparing polymer foams, attributed to its unique features including controllable cellular structure and exemption of toxic organic solvents^8,9^. Specifically, supercritical carbon dioxide (scCO_2_)^10,11^ is widely adopted as blowing agent for SCF due to its merits of non-toxicity, non-flammability, chemical stability, and less-demanding supercritical conditions (i.e., T_c_ = 31 °C, P_c_ = 7.38 MPa). Along with the rapid development of SCF technology, massive work has been conducted on the preparation of PVA foams. However, PVA shows poor foamability and exhibits an extremely narrow processing temperature range due to its strong hydrogen bonding. The solubility of CO_2_ in PVA depends on its moisture content, and only samples with a high moisture content can foam with CO_2_^12^. This is because the moisture in PVA acts as a plasticizer and enhances the foamability of PVA using scCO_2_ the as blowing agent. A PVA/microfibrillated cellulose (MFC) composite was successfully foamed using water and scCO_2_ as the co-blowing agents through continuous extrusion foaming^13^ and batch foaming^14^. The added MFC and the crystals that develop around them played a critical role in PVA/MFC composites foaming. A facile strategy based on complexation and plasticization between water and PEG molecules was designed by the authors to initiate the thermal processing of PVA^15–17^. Further, we prepared the foamed PVA materials with microcellular structure by using thermal processing and SCF technology^18–20^. Water, serving as the major plasticizer for PVA, can efficiently control its supramolecular structure and confine its crystallization. As a result, the foamability of plasticized PVA was substantially enhanced by the increased CO_2_ solubility. Unfortunately, the prepared PVA foams via thermoplastic foaming will always be the foams with closed-cell structure, which is not suitable for the diffusion of nutrients and growth of new tissue. Therefore, the main objective of this paper is to create the open-celled porous structure in PVA-based foams based on our previous work.

Poly (ethylene glycol) (PEG) is a water-soluble polymer and is available in molecular weights ranging from 200 to 20000. Due to its non-toxic, biodegradable and biocompatible properties, PEG is also widely applied in tissue engineering fields. PEG containing ether groups has an affinity for CO_2_ due to dipole-quadrupole interactions^21,22^, and the solubility of CO_2_ in PEG is much higher than that of polymer in most cases. For example, the solubility of CO_2_ in PEG is 1.8 times as large as that in polystyrene at 10 MPa and 110 °C^23^. Additionally, PEG presents similar polarity and structure to PVA, providing a miscible or partially miscible blend after the melt process. In view of these advantages, PEG can be considered as a good candidate for improving the thermoplastic foaming of neat PVA; thus, the porosity of the PVA scaffold can be controlled by blending various amounts of PEG. To the best of our knowledge, few results associated with PVA/PEG scaffold have been reported, and also the study on the thermoplastic foaming behaviour of PVA/PEG blend with scCO_2_ as blowing agent has not been reported to date.

In this study, the porous PVA/PEG blend scaffold was prepared through thermoplastic foaming using scCO_2_ as the physical blowing agent. The effect of PEG on the morphology and foaming behaviour as well as the interactions among the components, water state and thermal properties of PVA/PEG blends were systematically investigated. Due to the different solubility and diffusivities of scCO_2_ in PVA and PEG, the scaffold of the PVA/PEG blend showed a unique bimodal cellular structure, in which the smaller cells formed in the PEG phase were embraced in larger cells formed in the PVA phase. In addition, *in vitro* culture of the L929 fibroblast cells on the WPVA/PEG was performed to evaluate their cell viability with the CCK-8 assay, as it may have potential applications in the field of tissue engineering.

## Results

### Thermal performance of WPVA/PEG composites

**Figure 1:**
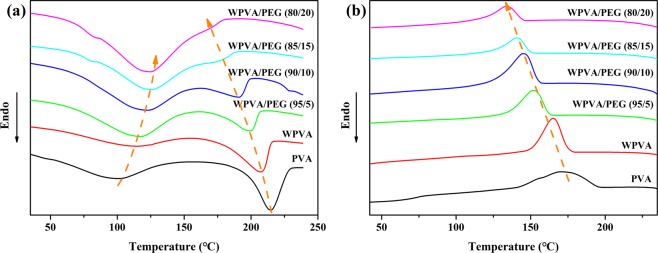
DSC curves of PVA, WPVA and WPVA/PEG-200 composites: (**a**) heating curves and (**b**) cooling curves.

PVA is a typical multi-hydroxyl polymer with strong intra- and intermolecular hydrogen bonding, leading to a relatively high melting temperature, very close to its decomposition point. Thus, the critical issue to realize the thermal processing of PVA is destroying its original hydrogen bonding and controlling its supermolecular structure to decrease its melting point. Herein the DSC curves were recorded to study the influence of PEG on the melting and crystalline behaviour of PVA. Figure 1 presents the heating and cooling DSC curves of dry PVA, water-plasticized PVA (WPVA) and WPVA/PEG composites with various PEG-200 (PEG with an average molecular weight of 200 g/mol) contents. The related data are summarized in Table 1. Two obvious endothermic peaks can be observed in the heating curves of all samples, in which the former peak is attributed to the evaporation of residual water and the latter one belongs to the melting of PVA. Apparently, the samples of WPVA and WPVA/PEG exhibit notably higher water evaporation temperature (T_p_) compared to bulk water (approximately 100 °C). This is because the interaction of hydrogen bonds between water and PVA molecules is stronger than that among sole water molecules. For the WPVA/PEG system, extra hydrogen bonds of PEG-water are generated; thus, more energy is needed to break these hydrogen bonding before evaporation. As expected, neat PVA shows a high melting temperature (T_m_) at 215 °C. Although removing the residual water in the WPVA sample during the first heating cycle, the plasticization effect of water on PVA chains is still preserved at high temperature, leading to a decrease in the melting point of WPVA. With the addition of PEG, the melting peaks of WPVA/PEG composites become weaker and broader, and gradually shift to a lower temperature, from 199 to 171 °C. This is due to the generated hydrogen bonding of PVA-PEG, disrupting the regular structure and improving the movement of PVA chains. Figure 1b reveals that WPVA/PEG composites tend to crystallize at lower temperatures compared to pure PVA, whose crystallization temperature (T_c_) is approximately 170 °C. In particular, T_c_ of the composite with 20 wt.% PEG decreases to a temperature as low as 134 °C. Obviously, the introducing of PEG into PVA matrix significantly disturbs the regularity of molecular chain arrangement in PVA upon cooling, thus hindering its crystallization.

**Table 1:** Related data concluded from the DSC curves of PVA, WPVA and WPVA/PEG composites with various PEG-200 contents.

Sample	T_m_/°C	T_p_/°C	T_c_/°C	T_g_/°C
PVA	215	100	170	82
WPVA	208	112	165	—
WPVA/PEG(95/5)	199	116	152	52
WPVA/PEG(90/10)	191	121	145	26
WPVA/PEG(85/15)	178	125	141	—
WPVA/PEG(80/20)	171	126	134	23

**Figure 2:**
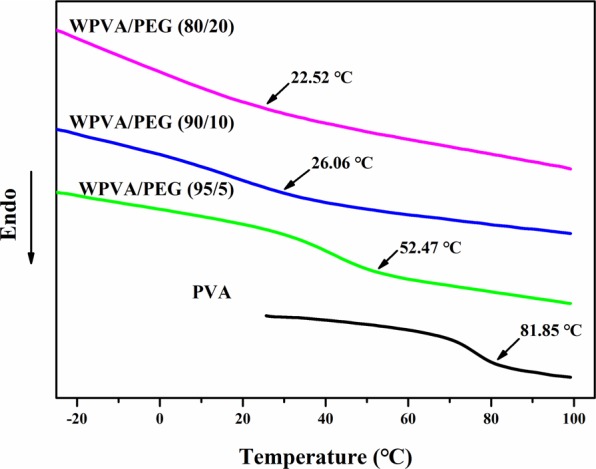
Glass transition temperatures of PVA and WPVA/PEG-200 composites.

In addition, DSC curves during the second heating cycle also provide the glass transition temperature (T_g_) of WPVA/PEG samples, which can effectively reflect the influence of PEG on the chain mobility of PVA. As clearly shown in Fig. 2, a T_g_ of 82 °C is detected for pure PVA. For WPVA/PEG composites, their T_g_s are significantly lower, and show a continuous descent with the rising of PEG content, for example, only 23 °C for the WPVA/PEG composite with 20 wt% PEG. This is also due to the fact that the original hydrogen bonds among sole PVA molecules were partially dismissed and re-formed among PVA and PEG molecular chains.

### Intermolecular interactions of plasticized PVA/PEG composites

**Figure 3:**
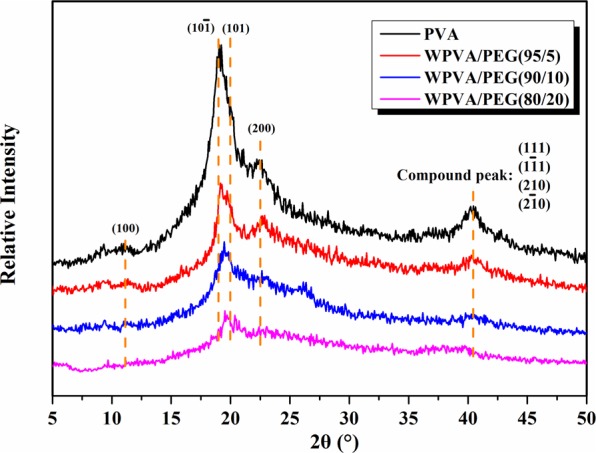
XRD patterns of neat PVA and WPVA/PEG composites with various PEG-200 content.

The influence of PEG on the crystalline structure of PVA was further studied by X-ray diffraction, as shown in Fig. 3. PVA, as a semi-crystalline polymer, exhibits five typical diffraction peaks in the angular range of 10°–50°, i.e., diffraction peak of plane (100) at 11.5°, (10$\bar{1}$) at 19.5°, (101) at 20.1°, (200) at 23.0°, and a compound peak of crystalline planes of (111), (1$\bar{1}$1), (210), and (2$\bar{1}$0) at around 40.5°^24,25^. As revealed, all these diffraction peaks for WPVA/PEG composites are weaker in intensity compared to pure PVA, and the intensity continuously declines with increasing PEG content, suggesting that the crystallization behavior of PVA chains is hindered by the plasticization between water and PEG. In general, the (101) diffraction is associated with the intermolecular interference among PVA chains and will provide useful information about the feature of hydrogen bonds. The enlarged (101) interplanar spacing suggests the weakened self-hydrogen bonding between PVA chains by generating the hydrogen bonding with water and PEG molecules. Thus, the reduced degree in crystallization is in favor of the melt processing of PVA.

**Figure 4:**
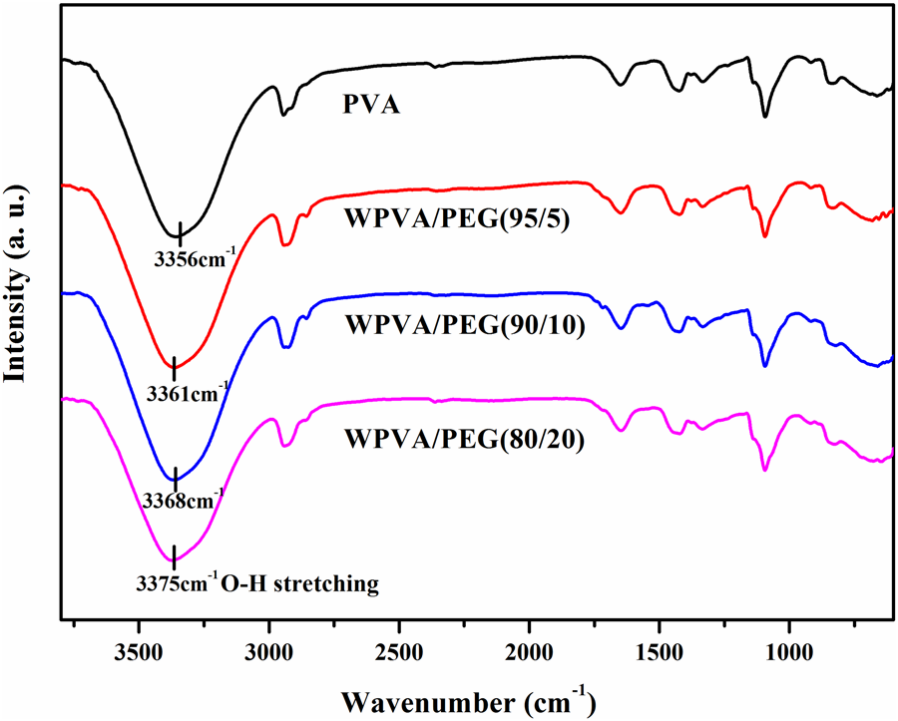
FTIR spectra of PVA and WPVA/PEG composites with various PEG-200 content.

**Figure 5:**
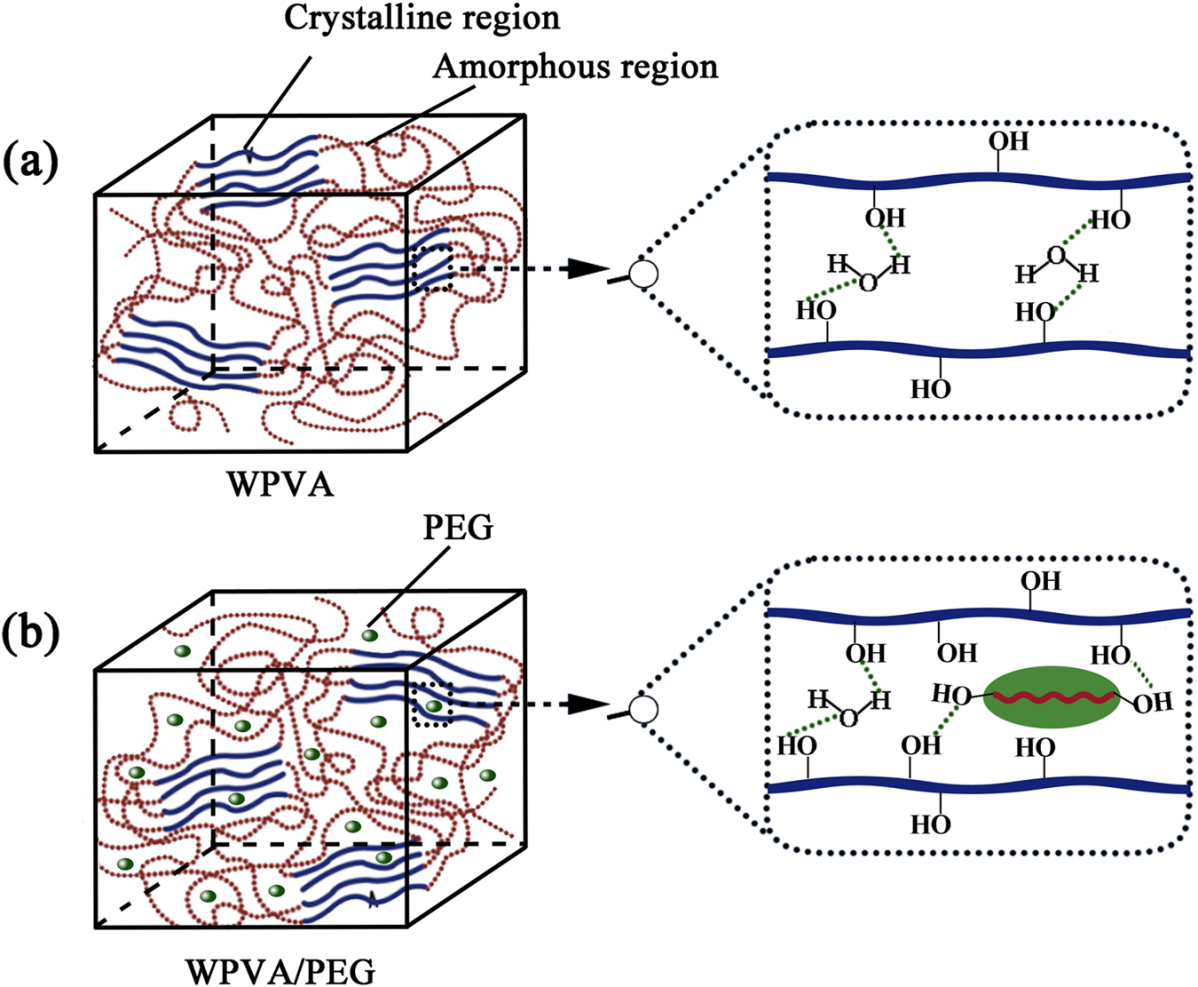
Scheme of hydrogen bonding in (**a**) WPVA and (**b**) WPVA/PEG.

To further interpret the interaction of complexation between PVA and PEG molecules, we conducted FTIR analysis so that various functional groups of PVA and WPVA/PEG samples can be identified. The corresponding FTIR spectra are presented in Fig. 4. As shown, neat PVA exhibits a considerably wide band of hydroxyl stretching spanning 3000–3800 cm^−1^, ascribed to the free alcohol (unbound -OH stretching band at 3600–3650 cm^−1^) in amorphous phase and the bonded hydroxyl (3200–3570 cm^−1^) in crystalline phase. Whereas, such bands of WPVA/PEG composites shift to higher wavenumbers, and the level of the blue-shift becomes larger for the composite with higher PEG content, for example, by approximately 19 cm^−1^ with the PEG content increasing to 20 wt%. As we all know, the hydrogen bonds in the system will always have a great impact on the stretching band of hydroxyl^26,27^. PVA is known to form hydrogen bonding among its OH groups both intermolecularly and intramolecularly, while WPVA/PEG composites can form extra hydrogen bonding among the OH groups from PVA chains and the PEG molecules, and the extra hydrogen bonding increases in the blend system with increasing PEG ratio, which can be illustrated by Fig. 5.

### Phase morphology of PVA/PEG composites

**Figure 6:**
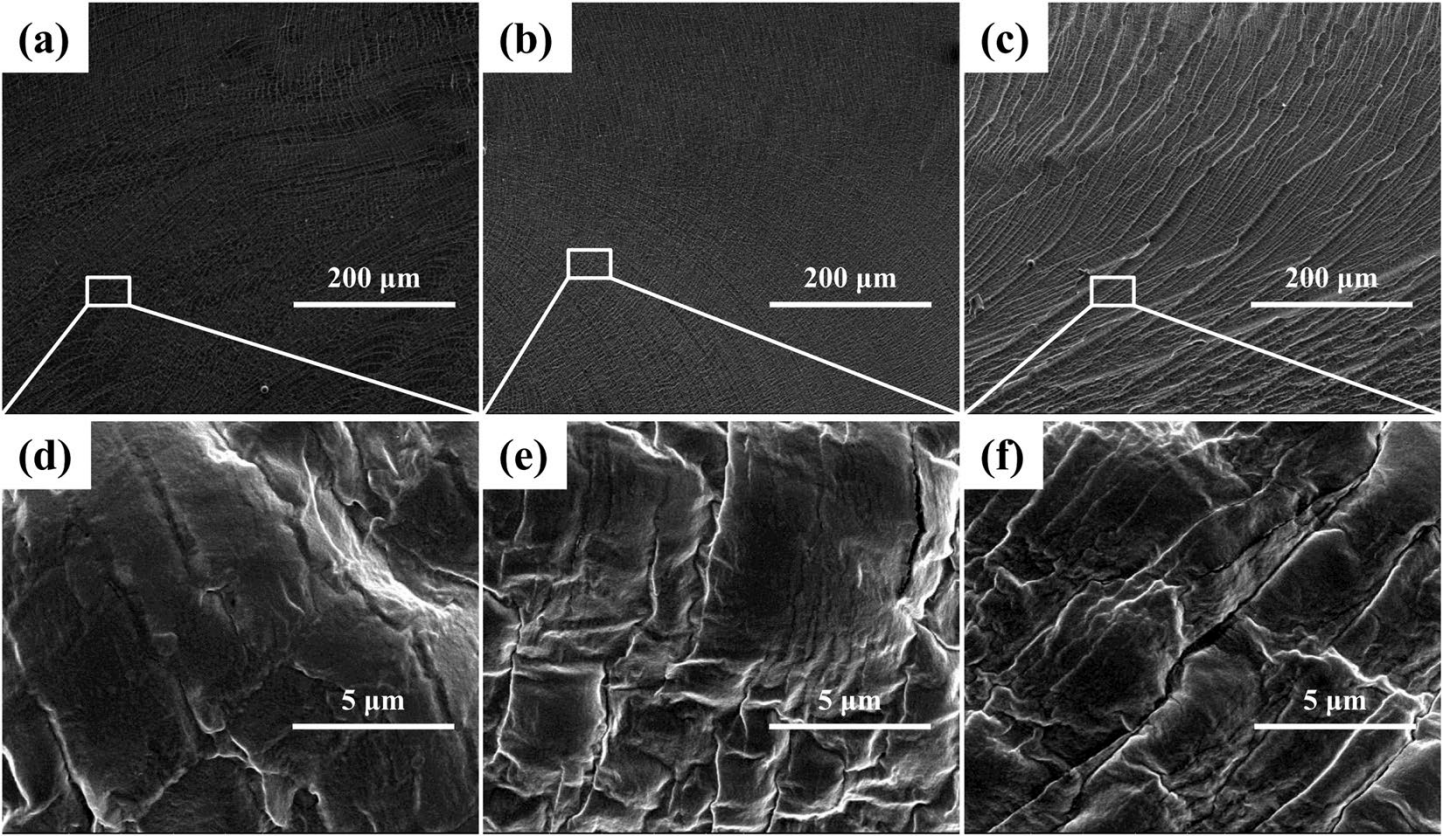
Micrographs of solid WPVA/PEG-200 blends: (**a**,**d**) WPVA/PEG (95/5); (**b**,**e**) WPVA/PEG (90/10) and (**c**,**f**) WPVA/PEG (80/20).

In general, the cellular structure created in the final foamed samples will always be affected by the phase morphology of a blended system. Herein, the micrographs of solid WPVA/PEG blends are investigated by SEM and the related images are shown in Fig. 6. Limited to the solution process of pure PVA, few studies reported the microstructure of PVA/PEG in the solid state. It is worth noting that we successfully obtained the phase morphology of WPVA/PEG through thermal processing. It is observed that a uniform and smooth surface morphology is found in the case of the WPVA/PEG blend with a low PEG-200 concentration (below 10 wt%). This is because a similar group and structure are observed in PVA and PEG chains. When the PEG concentration is further increased to 20 wt%, an obvious folded and rugged structure occurs on the fracture surface of the WPVA/PEG blend. PEG has a much larger chain length compared with water, and shows a limited compatibility with PVA. This means that the blended PVA/PEG systems are miscible only when the PEG-200 content is less than 10%. Thus, a different phase morphology caused by weak phase separation is detected in the WPVA/PEG blend with a high PEG concentration, like the immiscible WPVA/PEG (80/20) sample shown in Fig. 6f.

**Figure 7:**
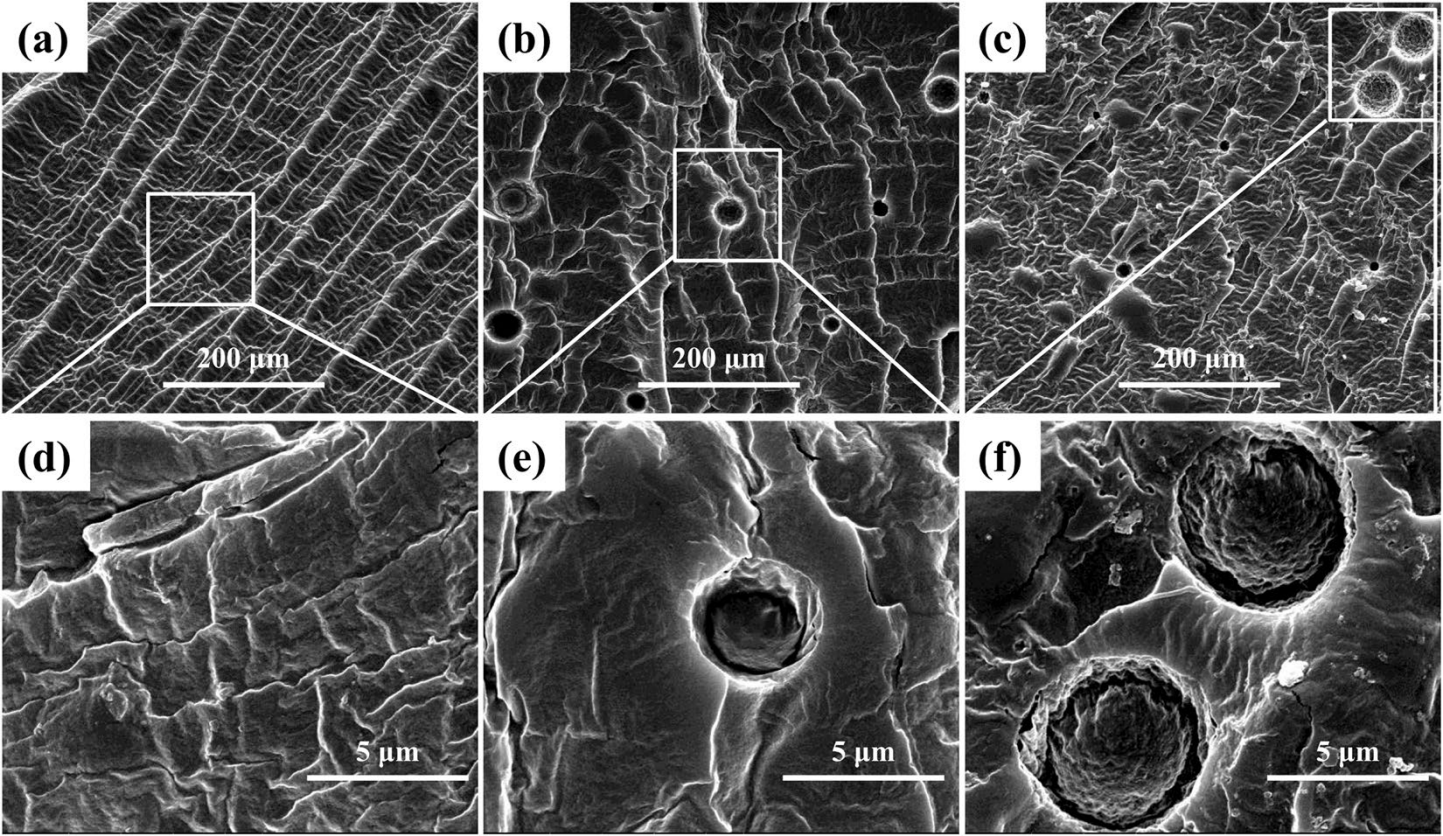
Micrographs of solid WPVA/PEG-6000 blends: (**a**,**d**) WPVA/PEG (98/2); (**b**,**e**) WPVA/PEG (95/5) and (**c**,**f**) WPVA/PEG (90/10).

PEG is available in a large range of molecular weight from 200 to 20000 g/mol. PEGs up to a molecular weight of 600 g/mol are liquid, while those with higher molecular weight are solid. In addition, the hydroxyl group density decreases for PEG with a higher molecular weight. We also studied the phase morphologies of WPVA/PEG blends in which the molecular weight of solid PEG is 6000 g/mol. As expected, PEG-6000 presents totally different phase morphologies in WPVA/PEG blends, compared with PEG-200. Resulting from the poor mobility of its long chain and small number of hydroxyl groups, PEG-6000 shows a significantly reduced compatibility with PVA, and a serious phase separation can be observed in WPVA/PEG blends, as shown in Fig. 7. Thus, we can conclude that PEG-6000 is immiscible with PVA. The phase domain of PEG-6000 grew in size with increased PEG concentration, and the phase structure of WPVA/PEG-6000 gradually changed into the sea-island structure. In addition, an increase in the PEG weight fraction was related to the increasing number of islands.

### Cell morphologies of PVA/PEG composite scaffold

Normally, polymer blends will always present a different foaming behaviour compared to the single polymer. Possible reasons include the heterogeneous nucleation in interfacial regions, different solubility and diffusion coefficients of the foaming gas in each phases, and the various viscosity of the components. Herein, in order to tune the cell structure of the PVA scaffold, the second phase (the PEG phase) was introduced into the WPVA system. The phase morphologies of the WPVA/PEG blend can be effectively adjusted by varying the addition content or molecular weight of PEG, thus realizing the modification of the cellular structure of the composite scaffold.

**Figure 8:**
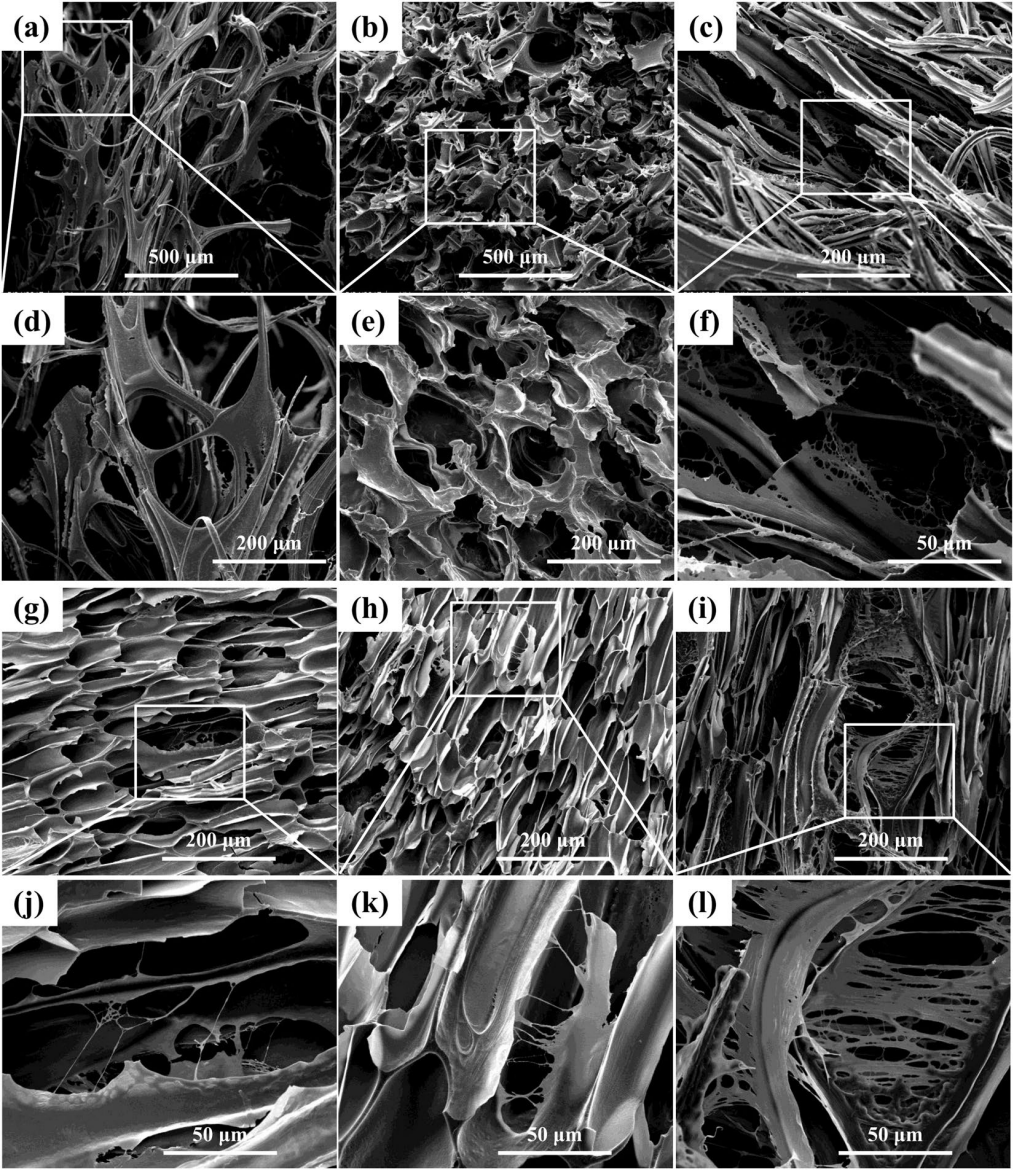
SEM micrographs showing the cellular structure of WPVA/PEG scaffolds: (**a**,**d**) WPVA/PEG-200 (95/5); (**b**,**e**) WPVA/PEG-200 (90/10); (**c**,**f**) WPVA/PEG-200 (80/20); (**g**,**j**) WPVA/PEG-6000 (98/2); (**h**,**k**) WPVA/PEG-6000 (95/5) and (**j**,**l**) WPVA/PEG-6000 (90/10).

In our previous studies^18–20^, the systematic experiments were carried out to investigate the SCF foaming of WPVA under different foaming conditions, including various foaming pressures, temperatures and saturation times. Therefore, the WPVA/PEG blends were foamed at the optimal condition of 120 °C, 12 MPa and a pressure release rate of 8 MPa/s. SEM micrographs of a foamed WPVA/PEG scaffold with various PEG contents and molecular weights were showed in Fig. 8. The foamed WPVA sample shows a uniform cellular structure after the rapid depressurization, even at relatively high foaming temperature^18^. However, after incorporating PEG, a significant change from closed-cell structure to open-cell structure is observed in the WPVA/PEG blend scaffold. Benefiting from the enhanced solubility of the blowing agent (CO_2_), the bubbles are easier to generate and grow in WPVA/PEG sample, thus leading to a highly porous open-cell structure with irregular cells. With increased PEG content, the WPVA/PEG (90/10) scaffold shows the increased number of cells with smaller cell size. This is because more dissolved CO_2_ in the polymer matrix will lead to an increase in the supersaturation of the polymer/gas system during the depressurization process. The energy barrier to nucleation strongly decreases, leading to more cells being nucleated with in a given volume, and thus more but smaller cells are generated. When the PEG content is further increased, with a sudden evolution of the phase morphology (as discussed above), a significantly different and unique cellular structure is created in the WPVA/PEG (80/20) sample. The scaffold has a special bimodal pore structure where the small-size pores are surrounded by the large ones.

In addition, we also investigated the foaming behaviour of the WPVA/PEG blend with high-molecular-weight PEG. The obtained cellular structure of the WPVA/PEG-6000 scaffold is shown in Fig. 8. As mentioned above, the phase morphology determines the final cellular structure created in the blend system. Due to the phase separation between PVA and PEG, the unique bimodal cellular structure is observed in all WPVA/PEG-6000 scaffolds. The number of small pores gradually increases with increased PEG concentration. Thus, it is easy to see that the small pores are created in the PEG phase, which is consistent with the phase morphology of the solid WPVA/PEG blend. Therefore, it is further confirmed that we can effectively adjust the cellular structure of the WPVA/PEG scaffold by varying the PEG concentration or the molecular weight of PEG.

**Figure 9:**
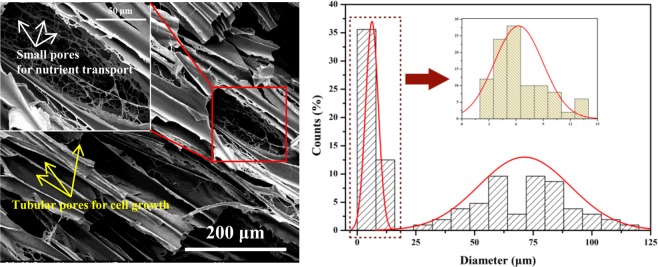
The bimodal cellular structure (the white arrows represent small pores for nutrient transport, and the yellow arrows represent tubular pores for cell growth) and the cell size distribution of the WPVA/PEG-6000 (90/10) scaffold.

As shown in Fig. 9, at the given processing parameters, the size range of the large cell is between 30 and 150 μm, while the range of the small cell is below 15 μm that is much smaller than that of neat WPVA foam. The foaming behavior of such a unique pore structure can be interpreted by considering the morphology and the diffusion behaviour of the physical blowing agent (scCO_2_) in each phase. On the one hand, WPVA/PEG blends with high PEG concentration or high-molecular-weight PEG present the sea-island structure because of the immiscibility between them. Furthermore, the solubility and diffusivity of CO_2_ in the “sea phase” (PVA phase) differ from those in the “island phase” (PEG phase). When high CO_2_ pressure is released, as a result of the higher solubility of CO_2_, the cell nucleation is enhanced in the PEG phase and a large number of small cells occur in the given volume. In contrast, fewer but larger cells are created in the PVA phase that embrace the small cells in the PEG phase. Specifically, a cellular structure with highly elongated pores is observed in the PVA phase. When used as the scaffold for tissue engineering, these tubular pores (as indicated by the yellow arrows) are beneficial to guide cell growth and extra cellular matrix deposition along the direction of the elongated pores arranged parallel to the surface/subchondral bone^1,28^. A large number of small pores (as indicated by the white arrows) with a diameter of approximately 10 μm are located at the pore wall surface, that is, the bimodal porous structures are interconnected, allowing for cell and nutrient transport throughout the scaffold^29,30^.

### *In vitro* cytotoxicity and cell adhesion behaviour

**Figure 10:**
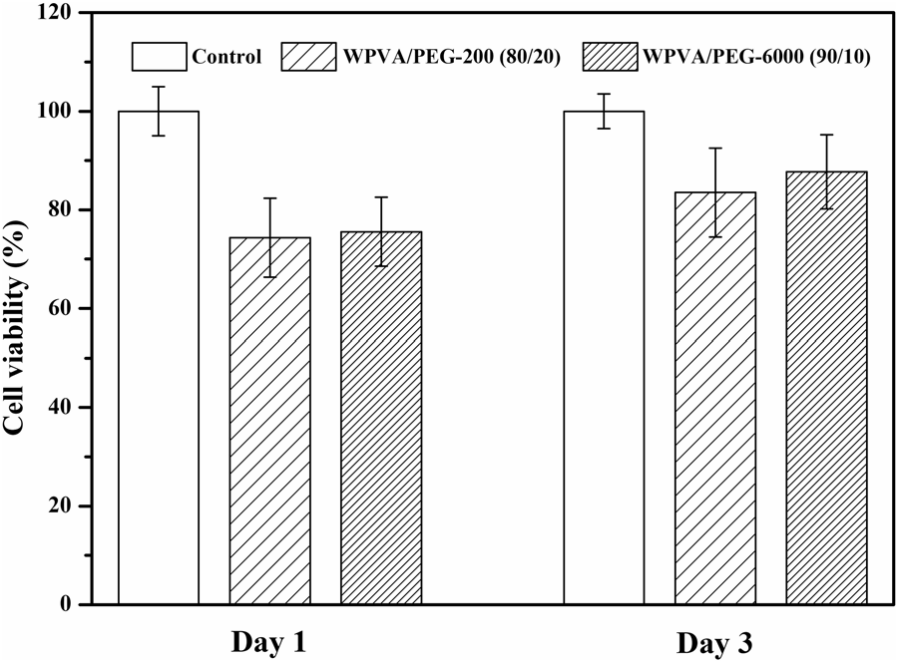
L929 fibroblast cells viability after 1 d and 3 d seeded on WPVA/PEG-200 (80/20) and WPVA/PEG-6000 (90/10) samples.

To assess the cytocompatibility of the WPVA/PEG scaffold, we performed *in vitro* culture of the L929 fibroblast cells on the WPVA/PEG samples and detected their cell viability with the CCK-8 assay. During tests, the WPVA/PEG sample should immerse in the DMEM. The cell viability of L929 fibroblast cells seeded on various WPVA/PEG samples is shown in Fig. 10. After 1 day, the cells seeded on the sample show slightly lower cell viability of approximately 75% due to the new environment the cell faces. After 3 days, the cell viability increases to above 80%, indicating that there is no cytotoxic effect on the L929 fibroblasts, compared with the cells cultured in the medium. The results indicate that the WPVA/PEG samples are nontoxic.

**Figure 11:**
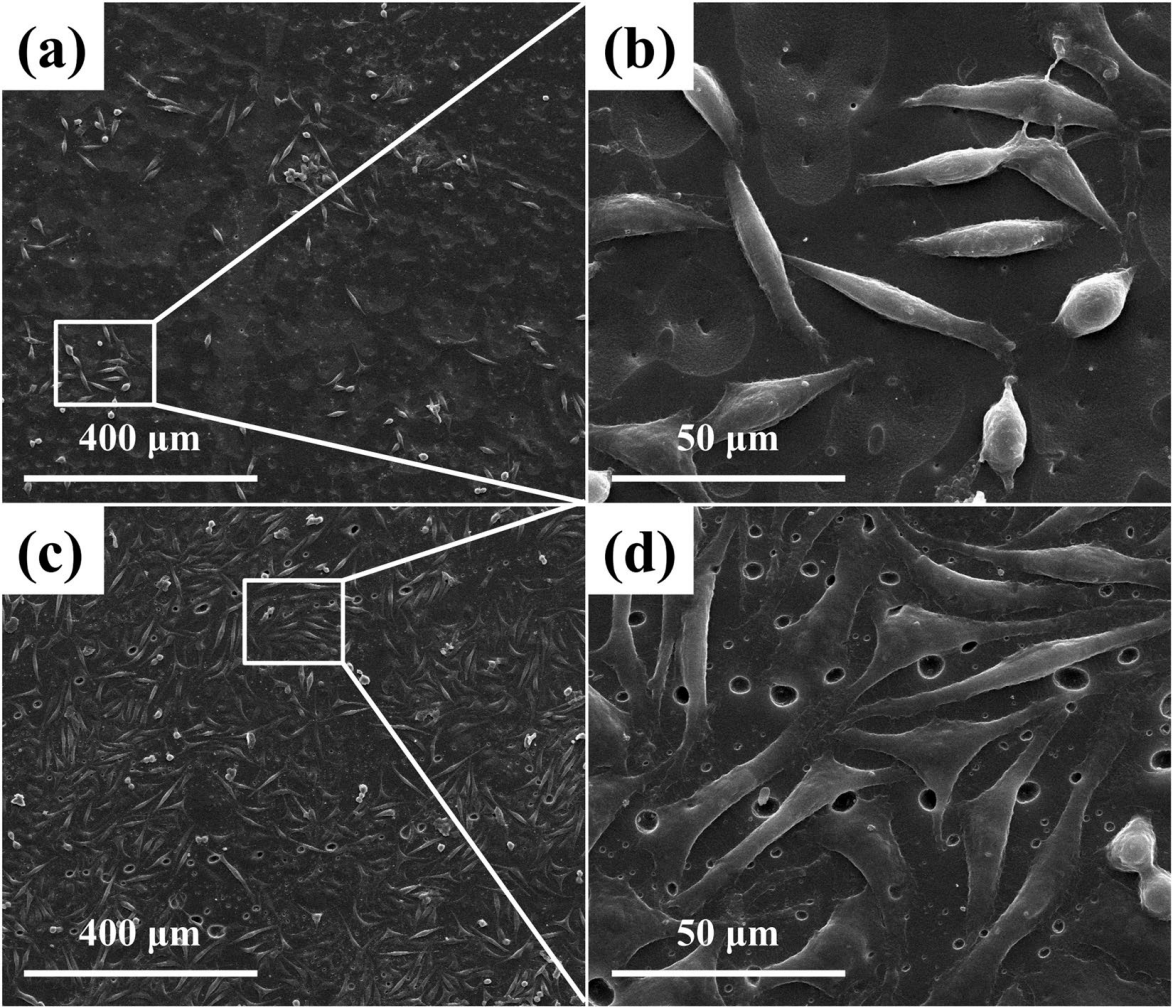
SEM images of cultured L929 cells on WPVA/PEG-6000 (90/10) samples after 1- (**a**,**b**) and 3-day (**c**,**d**) of incubation.

WPVA/PEG for soft tissue engineering applied scaffolds should have the ability to support adhesion and proliferation of cells. The cell culture results of the WPVA/PEG-6000 (90/10) sample evaluated by SEM are shown in Fig. 11. To illustrate cell growth on the material surface, the morphology of L929 cells after growth for 1 and 3 d was studied. SEM images demonstrate that the cells can attach to WPVA/PEG samples and grow very well. The L929 cells stretch out and tightly connect to the sample surface. The attached cells appear in the strap-like shape instead of the rounded morphology, indicating that the WPVA/PEG provides a favourable surface for cells to adhere. At the same time, with the timescale increasing from 1 to 3 days, the number of L929 cells on the WPVA/PEG sample obviously increases, and the surfaces were almost completely covered with L929 cells after a 3-d incubation. These results confirm that WPVA/PEG has good biocompatibility and facilitates cell adherence on it.

In summary, this study fabricated a novel WPVA/PEG scaffold by combining thermal processing and SCF technology. First, based on intermolecular interactions of complexation and plasticization, a wide thermoplastic foaming window for the WPVA/PEG blend was obtained. Then, the WPVA/PEG scaffold was successfully prepared using scCO_2_ as the physical blowing agent. WPVA/PEG blend scaffolds with high PEG concentration or high-molecular-weight PEG present a unique bimodal (large and small) open-celled structure with interconnected networks. This unique cellular structure is beneficial to cell growth and nutrient transport. In addition, WPVA/PEG has good biocompatibility and facilitates the L929 fibroblast cell attachment on its surfaces. Hence, these WPVA/PEG scaffolds with such a bimodal cellular structure may be suitable for tissue engineering applications.

## Methods

### Materials

Poly (vinyl alcohol) (PVA) (degree of polymerization is 1750 ± 50, hydrolysis degree is 99%) was provided by Sichuan Vinylon Works, SINOPEC (Chongqing, China). Raw PVA flakes were washed with water until it reached a pH of 7 to remove the residual impurities, and followed by drying process at 80 °C for about 2 h. Poly (ethylene glycol) (PEG) of 200 and 6000 g/mol was purchased from Chengdu KeLong Chemical Co. Ltd. (Chengdu, China). Carbon dioxide (physical blowing agent, 99.5%) was supplied by Taiyu Gas (Chengdu, China). L929 mouse fibroblast cells were obtained from the Internal Medicine Laboratory of HuaXi Medicinal Center (Chengdu, China). Dulbecco’s modified Eagle’s medium (DMEM) was purchased from Gibco Life (Grand Island, NY). Foetal bovine serum (FBS) was purchased from Hyclone. Deionized water was employed for all experiments.

### Preparation of WPVA/PEG composite

**Figure 12:**
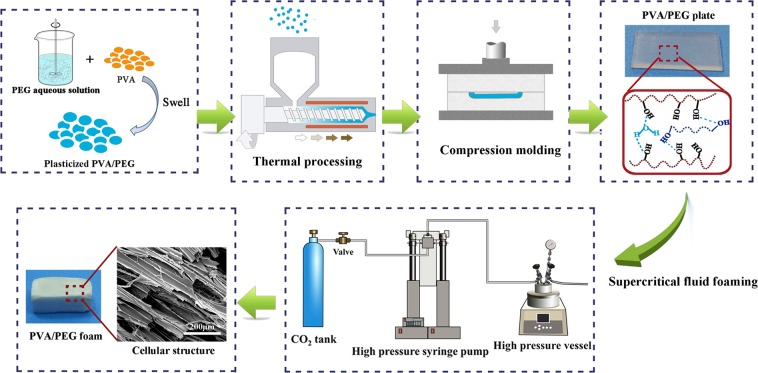
Schematic illustration of the process for fabricating the WPVA/PEG scaffold.

Quantified water and PEG were mixed to form a PEG aqueous solution under gentle mechanical stirring at room temperature. A certain amount of dried PVA was then added to the PEG aqueous solution, and the mixture was uniformly blended to let the solution completely swell into PVA at ambient temperature in a sealed container. As a result of the small molecular weight and favourable interactions with PVA, water will be able to easily diffuse into the PVA matrix, resulted in increased free volume and enhanced chain mobility, and also promoting the blending between PVA and PEG. According to our previous work^20^, the obtained water-plasticized PVA/PEG (WPVA/PEG) was melt processed through a single screw extruder (RM-200C, Harbin Harpro Electrical Technology Co., Ltd., Harbin) at the processing temperature range of 150–175 °C and cut into pellets. Then the plasticized prepared PVA/PEG composite pellets were moulded into plates (the thickness is 2.0 mm) using a plate vulcanization machine (under 165 °C, 15.0 MPa). Through the drying method, the water content of the WPVA/PEG samples was 27.5% by weight. Accordingly, the final PEG-200 (PEG-6000) content varied from 5% to10% and 20% (2%, 5% and 10%). The fabrication process is schematically shown in Fig. 12.

### Preparation of the WPVA/PEG composite scaffold

According to the foaming method of the plasticized PVA-based composite in our previous work^20^, the WPVA/PEG composite was the foam matrix and scCO_2_ was used as the physical blowing agent. First, the composite plates were saturated with scCO_2_ at constant temperature and pressure (the syringe pumps were purchased from Teledyne ISCO, Inc., USA) in a stainless-steel autoclave (Beijing Century Senlang Experimental Apparatus Co., Ltd.). After a fixed period of time, the supersaturated plate was produced when the pressure was removed (pressure-induced phase separation), resulting in the nucleation and growth of bubbles. The experiments were performed at the foaming temperature of 120 °C, saturation pressure of 15 MPa and depressurization rate of 8.0 MPa/s.

### Characterizations of solid WPVA/PEG blends

Thermal tests of various WPVA/PEG blends were performed on a differential scanning calorimetric apparatus (DSC, Q20, TA Instruments Co. Ltd., New Castle, USA) in a nitrogen atmosphere as described in our previous work^20^. The DSC tests of the WPVA/PEG blends were conducted from 40 °C to 250 °C to remove thermal history and cooled slowly to room temperature at a heating rate of 10 °C min^−1^ in the first cycle, and then the spectra were recorded at a heating rate of 5 °C min^−1^ in the second cycle, which showed similar procedure with our previous work^20^. The crystalline structure was investigated by a DX-1000 diffractometer (XRD, Dandong Fangyuan Instrument Co., Ltd., China). The CuKα generator system was operated at 40 kV and 25 mA, and the scanning 2θ ranged from 5° to 60° at a scanning rate of 1°/s. The Fourier transform infrared (FT-IR) spectra were obtained from a Nicolet 6700 FT-IR spectrometer (Thermo Nicolet Ltd, Vemon Hills, IL, USA). It should be noted that the XRD and FT-IR tests are designed on the basis of our previous work^20^. The phase morphologies of WPVA/PEG blends were investigated by scanning electron microscope (SEM), which was performed on an Inspect F field-emission electron microscopy (FEI, Eindhoven, Netherlands). Samples were fractured with liquid nitrogen and their surfaces sputtered with gold in a vacuum. Observations were taken at 0.5 Torr and 20 kV.

### Cellular structure of WPVA/PEG scaffold

To analyze the foam structure of the scaffolds, the obtained WPVA/PEG samples were dipped into liquid nitrogen and fractured, and then the cellular morphology of their fractured surfaces was investigated using SEM. Nano Measurer software and Origin were used to measure the cells diameters and to count their distribution as described in our previous work^20^.

### Cell culture

Cell culture was conducted according to the method in previous work^31^, and mouse fibroblast cells (L929) were cultured in DMEM supplemented with 10% FBS, 100 U/ml penicillin, and 100 μg/ml streptomycin at 37 °C and 5% CO_2_/95% air and kept at approximately 90% relative humidity in culture bottles. The medium was renewed every 2 days. Cells in increased logarithmic phase were rinsed with sterilized PBS and then incubated in 0.25% trypsin for 3 min. Trypsinization was stopped by adding DMEM with 10% foetal bovine serum. Cells were centrifuged and resuspended in DMEM with 10% FBS and then diluted to a certain concentration (1 × 10^6^ cells/ml).

### Cell adhesion analysis

As described in previous publications^32,33^, prior to cell culture, WPVA/PEG samples were sterilized with gamma radiation using doses of 20 kGy and then immersed in PBS overnight in a 24-well plate. Then the liquid was removed from the wells, fibroblast cells were seeded on WPVA/PEG samples and controls (blank wells), incubated at 37 °C/5% CO_2_ and 100 humidity and fed with DMEM containing 10% FBS and 2% penicillin/streptomycin. All cell culturing was carried out in a laminar flow hood under sterile conditions. Cell viability was assessed using CCK-8, and the optical density (O.D.) was obtained at 490 nm using a microplate reader (Model 680, Bio Rad Corp.). The O.D. value of cells cultured on the WPVA/PEG was compared with the O.D. value of the control to obtain the cell viability after a 1- or 3-day incubation. Three repeats were carried out to acquire an average value for each sample, and a statistical analysis was conducted. Besides, after 1 or 3 d incubation, cell-seeded samples were rinsed with PBS and fixed with 2.5% glutaraldehyde, followed by dehydration through a series of ethanol washes and finally observed by SEM.
